# The Nation's Tribute. Importance of Locality

**Published:** 1918-10-12

**Authors:** 


### THE MATRONS' AND SISTERS' DEPARTMENT.

### THE NATION'S TRIBUTE.

### IMPORTANCE OF LOCALITY.

Nearly all the magnificent educational and benevolent endowments in the United Kingdom owe part at least of their existence to public spirit strongly tinged with local sentiment. The Londoner, like old Thomas Guy, has dreamed of creating a centre of good works in the midst of the streets he daily trod. Rich merchants, physicians, lawyers, ecclesiastics have shown throughout the course of our island's history how strongly local attachment have influenced them in the disposition of their goods. There is hardly a village, certainly no town in the kingdom, which does not possess endowments which testify to this persistent love of home dwelling in the hearts of keen business men.

In setting itself to the task of gathering from the nation a tribute worthy of those to whom it is to be offered, the College of Nursing wisely allows full scope to this natural and powerful instinct. First springs the impulse to aid nurses whom the donors know in daily life, some of whom perhaps have aided them personally. The next step is the patriotic impulse to benefit English, Scottish, Welsh, or Irish nurses. Then comes the wider outlook embracing nurses in every part of the British Empire. Last of all the sense of kinship which links the whole world together makes the progress of nursing among alien races, and in regions outside the Flag, a matter of concern to the united body. But the impulse to begin at home, to do first the thing which lies nearest to the hand and to do it well, is by far the most insistent and widely disseminated, and it is on this, as we have said, that the unrivalled benevolent foundations of this country have been laid.

Already striking instances have been given that public benevolence is specially inclined to flow into local channels where nurses are concerned. The latest example of this spirit was that given by Lady Cowdray at the recent meeting of the Aberdeen Centre. Keenly identified as she is with the general movement, the form her own contribution assumes is the local one. She takes pride and pleasure in providing a scholarship for an Aberdeen nurse in particular.

The actual endowments secured for the benefit of the nursing profession up to last year have not been numerous. The nation's gift to Florence Nightingale was personal. Had she bought an annuity with it for her own use all would have approved. The nation's offering to Queen Victoria, out of which grew the Jubilee Institute, was again of a strictly personal nature. Had Queen Victoria founded an orphanage with it or built a church no one could have objected. The principal fund subscribed for the benefit of nurses is that built up in connection with the Royal National Pension Fund for Nurses. The Junius S. Morgan Benevolent Fund is, we believe, by far the largest sum contributed by the public for the express benefit of nurses. The reason is not far to seek. There has been no recognised channel through which the money could be collected and turned to account for the benefit of nurses. Hospitals do not invite endowments to provide scholarships for their nurses. Their needs are too great in other ways, and till lately they have been far from realising the call for such endowments. So local gratitude for nurses, local sentiment spurred by actual needs, has had little scope. Such money as has been forthcoming for the advancement of nursing has flowed into the channel of district nursing associations, formed for the benefit of the poor rather than of the nurses; indeed, in some notorious instances, actually exploiting the nurses in order to do good *à bon marché*.

All this has been altered by the founding of the College, and by the swift and influential development of its Local Centres. No one who feels grateful to his nurse and the profession can now lack the means of showing his gratitude practically. All over the kingdom are rising Finance Committees, affiliated to the parent College, which are invoking subscriptions for objects immediately affecting the interests of nurses, and closely linked with particular towns and counties. Emulation is already beginning its healthy operations. What one locality does to-day, another achieves to-morrow and outdoes. With a continuous succession of nursing needs brought into the open so that all may see, comes in natural sequence the money to meet them. Quite apart from any special appeal for the Tribute, the College of Nursing, by the very fact of its establishment, and by its widening circle of Local Centres, has brought the public into touch with the nurses. The public begins to find out what nurses need in order to make them better, happier, more secure from misfortune. Donors have confidence that their gifts will be well applied, since in every locality they find a Finance Committee of able business men assisting nurses in the management of affairs at present beyond their powers. The results now that the public know the need, have the opportunity of supplying it, and have confidence in the good administration of the funds can hardly yet be imagined.

